# Supplementary material for: Polarization shaping of high-order harmonics in laser-aligned molecules
Source: Sci Rep. 2016 Dec 20;6:39295. doi: 10.1038/srep39295 (PMC5172357; doi:10.1038/srep39295)
Supplement: Supplementary Information [file srep39295-s1.pdf]

Polarization shaping of high-order harmonics in laser-aligned molecules: Supplementary

E. Skantzakis[1], S. Chatziathansiou[1,2], P. A. Carpeggiani[5], G. Sansone[3,4,5], A. Nayak[3], D. Gray[1], P. Tzallas[1,3], D. Charalambidis[1,2,3,*], E. Hertz[6], and O. Faucher[3,6,*]

[1]Foundation for Research and Technology-Hellas, Institute of Electronic Structure and Laser, P.O. Box 1527, GR-711 10 Heraklion, Crete, Greece
[2]Department of Physics, University of Crete, P.O. Box 2208, GR71003 Heraklion, Crete, Greece
[3]ELI-ALPS, ELI-Hu Kft., Dugonics tér 13, H-6720 Szeged Hungary
[4]Institute of Photonics and Nanotechnologies (IFN)-Consiglio Nazionale delle Ricerche (CNR), Piazza Leonardo da Vinci 32, 20133 Milano, Italy
[5]Dipartimento di Fisica Politecnico, Piazza Leonardo da Vinci 32, 20133 Milano, Italy
[6]Laboratoire Interdisciplinaire CARNOT de Bourgogne, UMR 6303 CNRS-Université Bourgogne Franche-Comté, 9 Av. A. Savary, BP 47870, F-21078 DIJON Cedex, France
[*]Correspondence and requests for materials should be addressed to O.F. (email: olivier.faucher@u-bourgogne.fr) or D.C. (email: chara@iesl.forth.gr)

ABSTRACT

Supplementary material for manuscript "Polarization shaping of high-order harmonics in laser-aligned molecules"

1 EUV reflective analyzer

The harmonic field polarized in the Oyz plan is written $\overrightarrow{E}_{\mathrm{H}}(0, E_y, E_z)$, with $E_y = |E_y|\exp(i\phi/2)$ and $E_z = |E_z|\exp(-i\phi/2)$, where ϕ is the dephasing between the two field components. The reflective EUV polarization analyzer consists on a combination of various polarization sensitive optical elements including the silicon wafer, the silver mirror, and the grating of the spectrometer. It induces along the y- (*i.e.*, p-polarization) and z-component (*i.e.*, s-polarization) of the electric field a complex amplitude $a_p = |a_p|e^{i\psi_p}$ and $a_s = |a_s|e^{i\psi_s}$, respectively. $\psi_p - \psi_s$ accounts for the eventual dephasing introduced by the analyzer. The action of the analyzer is characterised by the tensor

$$\overrightarrow{\overrightarrow{A}} = \begin{pmatrix} 0 & 0 & 0 \\ 0 & a_p e^{i\psi_p} & 0 \\ 0 & 0 & a_s e^{i\psi_s} \end{pmatrix} \tag{1}$$

After reflection on the analyzer, the HHG field writes $E_{\mathrm{a}} = \overrightarrow{\overrightarrow{A}}.\overrightarrow{E}$. The dependence of its intensity with respect to the orientation Φ of the analyzer is described by the Malus' law

$$\mathscr{I}\mathrm{a}(\Phi) \propto |a_p|^2 \left[|E_y|^2 \left(\cos^2\Phi + R\sin^2\Phi\right) + |E_z|^2 \left(R\cos^2\Phi + \sin^2\Phi\right) + |E_y||E_z|(R-1)\sin 2\Phi\cos\phi\right], \tag{2}$$

where $R = \left(\frac{|a_s|}{|a_p|}\right)^2$ is defining the extinction ratio of the analyzer.

The two amplitude components of the incident field can be obtained by measuring the signal for two orientations of the analyzer corresponding to $\Phi = 0$ and $\Phi = \pi/2$:

$$|E_y|^2 \simeq \frac{1}{(1+R)} \frac{\mathscr{I}_{\mathrm{a}}(\Phi=0) - R\mathscr{I}_{\mathrm{a}}(\Phi=\pi/2)}{\mathscr{I}_{\mathrm{a}}(\Phi=0) - \mathscr{I}_{\mathrm{a}}(\Phi=\pi/2)}, \tag{3}$$

$$|E_z|^2 \simeq \frac{1}{(1+R)} \frac{\mathscr{I}_{\mathrm{a}}(\Phi=\pi/2) - R\mathscr{I}_{\mathrm{a}}(\Phi=0)}{\mathscr{I}_{\mathrm{a}}(\Phi=0) - \mathscr{I}_{\mathrm{a}}(\Phi=\pi/2)}. \tag{4}$$

The IR field $\overrightarrow{E}_0$ delivered by the laser system is polarized along the horizontal y axis. A half-wave plate (HWP1) oriented at $45^\circ/2$ with respect to the y axis is used to convert the linear into a circular polarization. In order to balance the amplitude of the harmonic between its components E_y and E_z, a small ellipticity can be induced along the horizontal or vertical direction of the driving field. This is achieved by tilting HWP1 by a very small angle.

2 Energy measurement of the near-circularly polarized 9th harmonic

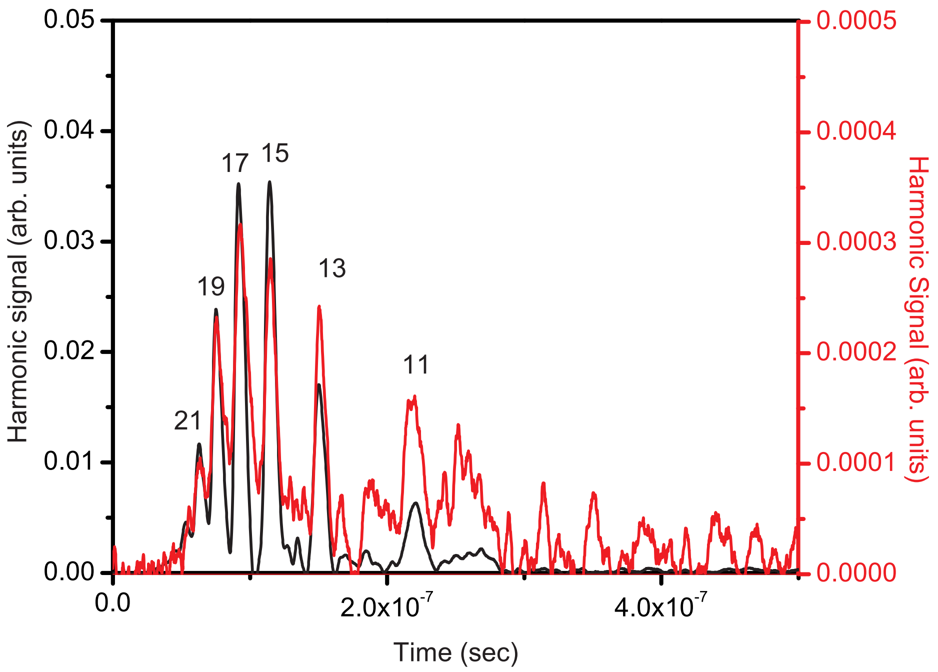

Figure 1. Photoelectron (PE) spectra resulted by the interaction of high-order harmonics with Ar (IP=15.76 eV). The black solid line shows the PE spectrum ($S^{(PE)}_{p\text{-pol.}}$) recorded in case of using only the p-polarized probe IR beam. In this case the energy of the EUV (E_{EUV}) has been measured by means of a calibrated EUV photodiode. The red line shows the PE spectrum ($S^{(PE)}_{\text{c-pol.}}$) recorded in case of using a p-polarized probe beam which results to the generation of a p-polarized 9th harmonic with an energy approximately equal to the energy of the near-circularly polarized 9th harmonic.

The energy of the near-circularly polarized 9th harmonic has been obtained by means of a calibrated EUV photodiode and a time-of-flight (TOF) photoelectron (PE) spectrometer, placed just after the EUV photodiode. The energy of the high-order harmonics (E_{EUV}) generated using only the p-polarized probe IR beam has been measured with the calibrated EUV photodiode, placed after the silicon plate and a 150-nm-thick aluminum filter, which transmits harmonics with order $\geqslant$ 11th. This was done by moving the silver mirror (Fig. 1 of the main text of the manuscript) out of the harmonic beam path. The corresponding PE spectrum ($S^{(PE)}_{p\text{-pol.}}$) induced by the interaction of the harmonics with order $\geqslant$ 11th with argon atoms was recorded (black line in Fig. S1) by moving the EUV photodiode out of the harmonic beam path. In this way the correspondence between the PE spectrum and the EUV energy has been established. A PE spectrum ($S^{(PE)}_{\text{c-pol.}}$) has been also recorded (red line in Fig. S1) after reduction of the energy of the 9th harmonic generated by the p-polarized IR beam to a value (signal recorded by the EUV spectrometer) equal with the energy of the near-circularly polarized 9th harmonic. This measurement gives the correspondence between the $S^{(PE)}_{\text{c-pol.}}$ spectrum and the energy of the near-circularly polarized 9th harmonic. With the above measurements the energy of the near-circularly polarized 9th harmonic just after the harmonic generation medium has been

estimated by using the relation

$$E_{9\text{th}} \approx C_q \frac{E^{(\text{ph})}_{\text{EUV}}}{Q T_{\text{Al}} R_{\text{Si}}} / \frac{S^{(\text{PE})}_{p\text{-pol.}}}{S^{(\text{PE})}_{\text{c-pol.}}}, \tag{5}$$

with Q (≈ 2), T_{Al} (≈ 5), R_{Si} (≈ 0.6), and C_q (≈ 0.2) being the quantum efficiency of the photodiode, the transmission of the Al filter, the reflectivity of the Si plate, and $C_q = S^{(9\text{th})}_{p\text{-pol.}} / S^{(\text{PE})}_{p\text{-pol.}}$ (where $S^{(9\text{th})}_{p\text{-pol.}}$ is the PE signal of the 9th harmonic), respectively. In the above calculation it has been assumed that the energy of the 9th harmonic is approximately equal with the energy of the 11th, 13th harmonics, *i.e.*, $S^{(9\text{th})}_{p\text{-pol.}} = S^{(11\text{th},13\text{th})}_{p\text{-pol.}}$. This approximation is valid since these harmonics are laying in the plateau region of the spectrum.
